# X-ray absorption spectroscopy and actinide electrochemistry: a setup dedicated to radioactive samples applied to neptunium chemistry

**DOI:** 10.1107/S1600577521011115

**Published:** 2022-01-01

**Authors:** Richard Husar, Thomas Dumas, Michel L. Schlegel, Daniel Schlegel, Dominique Guillaumont, Pier-Lorenzo Solari, Philippe Moisy

**Affiliations:** a CEA, DES, ISEC, DMRC, Université de Montpellier, Marcoule, France; b CEA, DES-Service d’Etudes Analytiques et de Réactivité des Surfaces (SEARS), CEA, Université Paris-Sacly, 911191 Gif-sur-Yvette, France; c ESTA–École Supérieure des Technologies et des Affaires, 90004 Belfort Cedex, France; d Synchrotron SOLEIL, L’Orme des Merisiers Saint-Aubin, BP 48, 91192 Gif-sur-Yvette Cedex, France

**Keywords:** X-ray absorption spectroscopy, XAS, actinide, electrochemistry, radioactive sample, neptunium chemistry

## Abstract

A spectroelectrochemical setup has been developed to investigate radioactive elements in small volumes (0.7 to 2 ml) under oxidation–reduction (redox) con­trolled conditions by X-ray absorption spectroscopy (XAS). The cell design is presented together with *in situ* XAS measurements performed during neptunium redox reactions. Cycling experiments on the NpO_2_^2+^/NpO_2_^+^ redox couple were applied to qualify the cell electrodynamics using XANES measurements and its ability to probe modifications in the neptunyl hydration shell in a 1 mol l^−1^ HNO_3_ solution. The XAS results are in agreement with previous structural studies and the NpO_2_^2+^/NpO_2_^+^ standard potential, determined using Nernst methods, is consistent with measurements based on other techniques. Subsequently, the NpO_2_^+^, NpO_2_^2+^ and Np^4+^ ion structures in solution were stabilized and measured using EXAFS. The resulting fit parameters are again compared with other results from the literature and with theoretical models in order to evaluate how this spectroelectrochemistry experiment succeeds or fails to stabilize the oxidation states of actinides. The experiment succeeded in: (i) implementing a robust and safe XAS device to investigate unstable radioactive species, (ii) evaluate in a reproducible manner the NpO_2_^2+^/NpO_2_^+^ standard potential under dilute conditions and (iii) clarify mechanistic aspects of the actinyl hydration sphere in solution. In contrast, a detailed comparison of EXAFS fit parameters shows that this method is less appropriate than the majority of the previously reported chemical methods for the stabilization of the Np^4+^ ion.

## Abstract 2

A spectroelectrochemical setup has been developed to investigate radioactive elements in small (0.7 to 2 ml) volumes under oxidation–reduction (redox) controlled conditions by X-ray absorption spectroscopy (XAS). The cell design is presented together with *in situ* XAS measurements performed during neptunium redox reactions.

## 1. Introduction

*In situ* X-ray absorption spectroscopy (XAS) in combination with electrochemical techniques is a powerful combination for determining the coordination of metal ions at a controlled oxidation state in various electrolytes (Nockemann *et al.*, 2009 ▸; Achilli *et al.*, 2016 ▸). Such an *in situ* approach is nowadays commonly applied for *d*-block elements and rare earth compounds, but is still very limited for actinide elements (An). These elements display unusual redox properties and complexation behaviour (Brown, 1978 ▸). Understanding the equilibria between oxidized and reduced species, the formation of inter­mediate unstable species and the magnitude of relevant redox couples under the conditions of inter­est is essential for predicting An chemistry in industrial and natural environments. Specifically, the characterization of inter­mediate species or unstable oxidation states is of fundamental inter­est in understanding how the inter­nal structures of An mol­ecular complexes affect their reactivity and the exchange between oxidation states.

To investigate these reactions at the mol­ecular scale using XAS, a spectroelectrochemical cell was designed at Argonne National Laboratory (USA) (Antonio *et al.*, 1997 ▸). The limited volume of this cell (5 ml) allowed for the characterization of radioactive samples using a limited amount of haza­rdous radionuclide with a concentration range (10^−2^ to 10^−4^ mol l^−1^) that corresponds to XAS measurements. Thus, this cell has been used to investigate An redox chemistry under acidic conditions (Soderholm *et al.*, 1999 ▸), alkaline conditions (Williams *et al.*, 2001 ▸) and on frontier elements such as berkelium (Antonio *et al.*, 2002 ▸). Subsequent XAS research monitoring actinide and radioactive elements coordination during *in situ* electroactive processes was performed with less active nucleides, such as U (Hennig *et al.*, 2005 ▸) or Tc (Antonio *et al.*, 2002 ▸; Poineau *et al.*, 2006 ▸). Although high-activity samples (Np and Pu) were used in several experiments in recent years, their combination with an electrochemical setup was hampered for various safety reasons. To contribute to this effort and to overcome these limitations, we have developed an electrochemical–XAS setup available for high-activity samples. The setup was designed taking into account specific requirements: (i) a small sample volume to limit radioactivity and permit the study of highly radioactive materials; (ii) the possibility to perform reproducible electrochemical reactions *in situ* (*i.e.* directly on the beamline) with highly radioactive material during an XAS experiment; (iii) (moderate) flexibility in the choice of working, reference and counter electrodes; (iv) easy access and handling to limit the risk of spillover during sample preparation; (v) tightness and number of barriers satisfying the synchrotron safety requirements for the handling of radioactive samples; and (vi) a small overall size to facilitate transport between the synchrotron facility and actinide laboratories. The cell has been successfully implemented on the MARS beamline at the SOLEIL synchrotron (LLorens *et al.*, 2014 ▸). The first set of experiments was performed in 1 *M* NHO_3_ to follow *in situ* structural and electronic changes of the neptunium ions under potentiometric control. The results demonstrate the capacities and limits of such a microcell setup and are discussed in comparison with purely Np electrochemistry results (*i.e.* laboratory scale and no X-ray techniques) (Cohen & Hindman, 1952 ▸; Cohen *et al.*, 1954 ▸; Takao *et al.*, 2009 ▸; Hindman *et al.*, 1958 ▸; Sornein *et al.*, 2009 ▸; Kihara *et al.*, 1999 ▸; Kim *et al.*, 2004 ▸, 2005 ▸; Cohen, 1961 ▸; Zielen *et al.*, 1958 ▸) or previous structural XAS studies (Bonin *et al.*, 2009 ▸; Allen *et al.*, 1997 ▸; Di Giandomenico *et al.*, 2009 ▸; Combes *et al.*, 1992 ▸; Reich *et al.*, 2000 ▸; Soderholm *et al.*, 1999 ▸; Hennig *et al.*, 2005 ▸; Williams *et al.*, 2001 ▸; Den Auwer *et al.*, 1999 ▸; Scheinost *et al.*, 2016 ▸; Antonio *et al.*, 1997 ▸, 2001 ▸, 2012 ▸; Ikeda-Ohno *et al.*, 2008 ▸, 2009 ▸).

While the main aim was to check the ability of the setup to proceed safely with the combination of electrochemistry and XAS measurements under confined conditions, a more critical evaluation of the structural results is also proposed. The systematic comparison of the structural EXAFS parameters extracted from (i) this work, (ii) formal works and (iii) theoretical calculations is proposed. This allows for a better understanding of the main benefits of such an electrochemical cell concept, but also some limitations that should not be ignored.

## 2. MARS electrochemical–XAS setup

**Figure 1:**
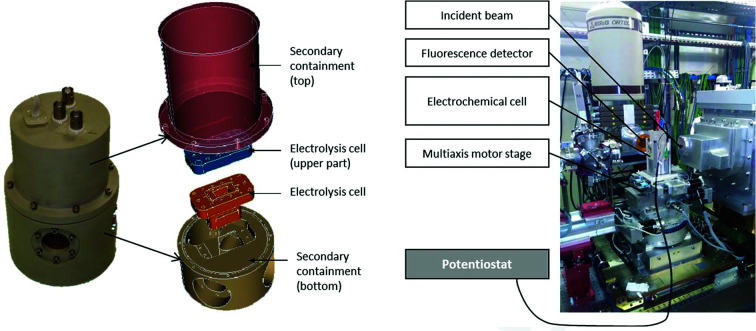
(Left) Double confinement electrochemical–XAS setup, 0.7 < *V*_sol_ < 2 ml (a magnetic stirrer is placed at the bottom of the setup). (Right) The electrolysis cell at the MARS beamline CX3 end-station.

The setup was designed to facilitate electrochemical control of a limited volume (700 to 2000 µl) of radioactive solution or suspension while at the same time guaranteeing double confinement of these haza­rdous samples and allowing relatively easy handling in the laboratory and on the beamline. The setup was made of an inner cell and an outer envelope (Fig. 1 ▸). The inner cell is made of PEEK, a material that is relatively inert, and thus can be used for acidic, basic and even non-aqueous solutions compatible with this material (such as room-temperature ionic liquids and organic solutions like dodecane or hepta­ne). Two X-ray windows are obtained by locally thinning the inner cell walls down to 200 µm. This design limits the risk of leaking at the X-ray windows, while providing a moderate X-ray attenuation (95% of the X-ray flux transmitted at 17 keV). This cell is enclosed in a secondary containment, also made of PEEK, to prevent dispersion in case of inner cell failure. The inner cell is rotated at 45° with respect to the incident beam, and three windows made of 90 µm Kapton film sealed with three screwed clamping rings to the second envelope provide paths for the incident beam, the transmitted beam and the fluorescence signal. The path length of the transmitted X-ray beam in the inner cell is about 11 mm.

The volumes of liquid that can be introduced in the inner cell vary between 750 (the minimum volume to soak up the windows) and 2000 µl. The bulk solution is steered (600 rpm) by a magnetic bar driven by a stirrer located outside the second envelope. Bulk electrolysis is then performed in the inner cell.

The electrodes are screwed and glued onto the lid of the inner cell to further limit possible leaking of fluids. The nature and distribution of electrodes can be tailored to meet the need of a specific experiment. For example, the working electrode can be made of platinum or any other metallic material, or even carbon. Our conventional reference electrode is an Ag/AgCl microelectrode (World Precision Instruments). How­ever, any other microelectrode could in principle be used, provided it can fit through the inlet (4 mm in diameter) and be reasonably short (a few cm) and sturdy. In a more recent experiment, a homemade reference electrode dedicated to room-temperature ionic liquids was also used in the same cell (Bengio *et al.*, 2018 ▸, 2020 ▸). The counter-electrode can plunge directly into the solution or, alternatively, it can be isolated from the main solution by a tube closed by a porous frit acting as a salt bridge. This setup somewhat hinders parasitic reactions on the electrode surface which might disturb the desired reaction of actinide redox transformation.

The setup performance was assessed by experiments of Np redox in 1 *M* HNO_3_. The working electrode is a platinum coiled electrode and the auxiliary electrode is Pt wire separated from the buck by a tube closed by a porous frit. The reference electrode is a 2 mm-diameter Ag/AgCl microelectrode from World Precision Instrument. Preliminary tests with the counter-electrode bathed directly in the experimental solution failed to achieve qu­anti­tative oxidation (or reduction) up to the target oxidation state although the target potentials had been validated previously by spectroelectrochemical UV–Vis absorption spectroscopy. Instead of a progressive change in oxidation state under the applied conditions, competitive side reactions seems to occur and are manifested by important current flow but no significant changes in redox speciation. For the later experiments, the counter-electrode was placed in the frit-sealed tube, which resulted in a reproducible experiment that is presented in the following results.

## 3. Experimental

### 3.1. Sample preparation

The sample preparation was performed in a radiochemistry laboratory at the ATALANTE facility (CEA Marcoule, France) in a dedicated glove-box. The purity of the oxidation state of the Np^V^ stock solution was checked with a UV–Vis spectrometer (Carry). A sample of 0.1 m*M* Np^V^ was transferred to the inner cell *via* a dedicated holder. The filling hole was then closed with a screw and sealed with ep­oxy glue. Basic tests were performed in the laboratory to check for a correct electrochemical behaviour of the cell. The second envelope was then closed and the cell can be easily shipped due to its relatively small size.

### 3.2. XAS experiment

The electrochemical cell was installed on the CX3 station of the MARS beamline, which is dedicated to XAS (Jeanson *et al.*, 2009 ▸; LLorens *et al.*, 2014 ▸). A 13-element HPGe solid-state detector (ORTEC) was used to collect the Np signal in fluor­escence mode. The energy calibration of the mono­chrom­ator was performed at the yttrium *K*-edge. All measurements were performed at room temperature.

### 3.3. Bulk electrolysis

Bulk electrolysis was performed over a period of 30 min using a remote-controlled potentiostat (μAutolab, Metrohm) located in the experimental hutch. Chrono-amperometric sequences of 30 min were alternated with acquisition of XAS data. It is worth mentioning that shorter electrolysis times or simultaneous electrolysis–XAS acquisition may artificially suggest hysteresis on Np redox couples under the chosen conditions, a consequence of incomplete or ongoing elec­trol­ysis. Moreover, the recorded chronoamperogram, even indicating the exponential shape expected for dilute solution electrolysis, never reached a null current during the experiment, indicating parasitic reactions.

Data processing was carried out using the *Athena* code (Ravel & Newville, 2005 ▸). The *E*_0_ energy was set at the maximum of the absorption edge. The EXAFS signal was extracted by subtracting a linear pre-edge background and a combination of cubic spline functions for the atomic absorption background and for normalizing the signal by the Lengeler–Eisenberg procedure. Fourier transforms (FT) were obtained by Fourier transform in *k* ^3^χ(*k*) between 2.5 and 11 Å^−1^. Selected FT contributions were fitted in *R*-space over individual radial distances and Debye–Waller factors (σ^2^) for every considered distance, using backscattering amplitude and phase shift functions obtained with *FEFF8.2* (Rehr & Albers, 2000 ▸) performed on structures optimized by density functional theory (DFT) calculations (see §4, *DFT calculations*). The amplitude reduction factor *E*_0_^2^ was set at 0.9. All fitting operations were performed. The *R* factor (%) and errors in distances were provided by *ARTEMIS* (Ravel & Newville, 2005 ▸).

## 4. DFT calculations

The geometry and frequency calculations were performed with *GAUSSIAN16* (Frisch *et al.*, 2016 ▸) at the DFT level of theory. A small core quasi-relativistic effective core potential (RECP-60 electrons) (Cao & Dolg, 2004 ▸; Küchle *et al.*, 1994 ▸) by the Stuttgart–Cologne group and its corresponding TZ-valence basis set were used for the neptunium ion. The PBE0 functional was used with the def-TZVP (Schäfer *et al.*, 1994 ▸) basis sets for O and H atoms. Aqueous solvation effects were taken into account using two explicit hydration shells. Effects beyond the second hydration shell were described through an implicit solvation model. The Integral Equation Formalism Polarizable Continuum Model (IEFPCM) was used as implemented in *GAUSSIAN16*.

The *ab initio* Debye–Waller factors (σ^2^) were calculated at 300 K for each scattering path from the dynamical matrix extracted from the DFT frequency calculations with the DMDW module of *FEFF9* (Rehr *et al.*, 2010 ▸; Rehr & Albers, 2000 ▸).

## 5. Results

### 5.1. Np^VI^/Np^V^

**Figure 2:**
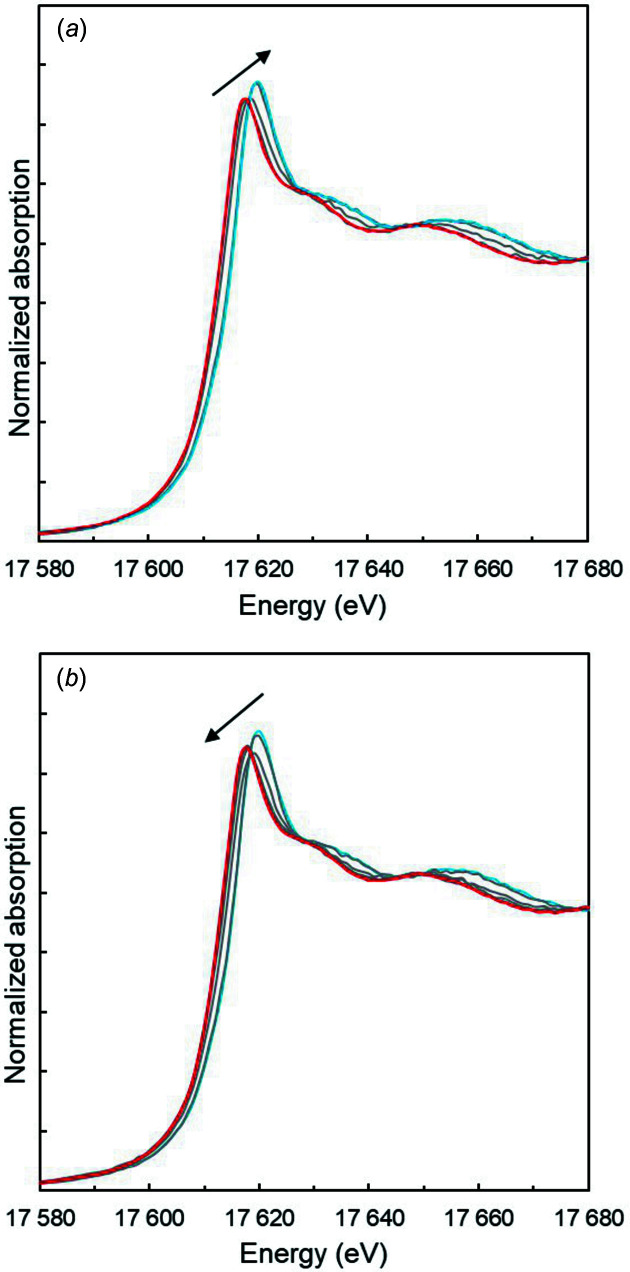
Normalized Np *L*_3_-edge XANES spectrum for (*a*) oxidation and (*b*) reduction experiments. The red spectrum is the pure Np^V^ XANES spectrum and the blue spectrum is the pure Np^VI^ XANES spectrum. The grey spectrum resamples inter­mediate states.

The first electrolysis experiment was performed with an initial Np^V^ solution. XANES and EXAFS spectra were recorded after 30 min of electrolysis for each potential step at 850, 950, 975, 1000, 1025 and 1150 mV/(Ag/AgCl) in the oxidation (anodic) direction, and at 1150, 1050, 975, 950, 925, 900, 875, 800 and 750 mV/(Ag/AgCl) in the reduction (cathodic) direction (Fig. 2 ▸). All XANES spectra display a white line, which is shifted from 17616 to 17619.5 eV as Np^V^ is oxidized to Np^VI^. The high-energy shoulder of the absorption edge, located near 17628 eV and attributed to multiple scattering in the neptunyl moiety, is also shifted to higher energy, *i.e.* 17634 eV, consistent with a *trans*-dioxo structure for both +V and the +VI oxidation states. Conversely, a shift of the white line and of the shoulder back to their initial values is measured during the reduction steps of Np^VI^ back to Np^V^. From Np^V^ to Np^VI^, the white line increases in intensity. An isosbestic point at 17617.7 eV repeats itself in both the oxidation and the reduction sequences, suggesting that only two components were simultaneously present in solution for each sequence. Transitional spectra were reproduced by linear combination fits using the two extrema spectra for Np^V^ and Np^VI^, resulting in Np^IV^/Np^V^ ratios for each potential. As pointed out by Soderholm *et al.* (1999 ▸), a direct estimation of the redox species is only possible if there is no change in the experimental setup during the data acquisition except for the imposed potential in the solution.

**Figure 3:**
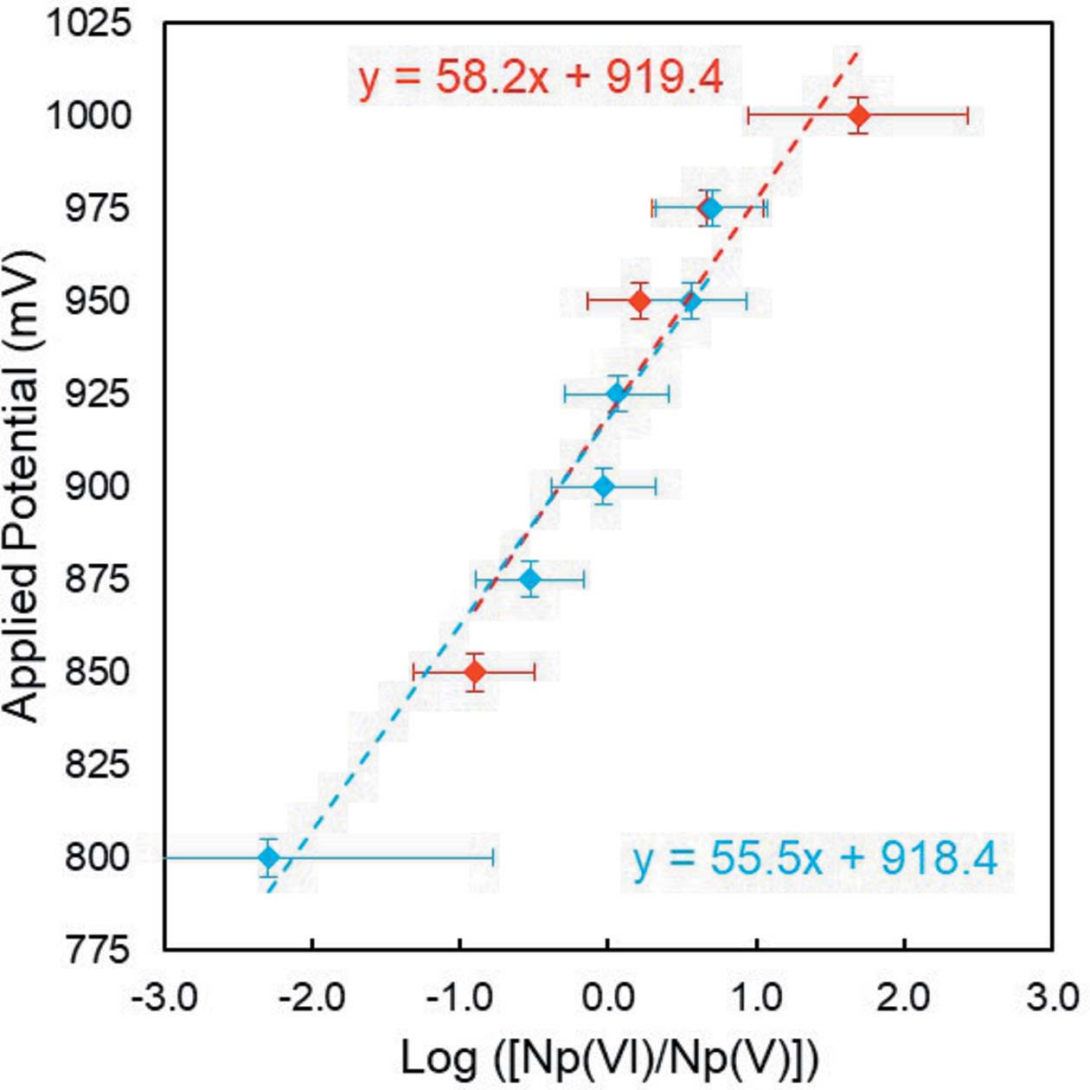
Nernst plots obtained from XANES measurements. Red diamonds cor­respond to the oxidizing experiment and blue diamonds to the reducing experiment. The dashed lines are the linear regression.

The relative Np^VI^/Np^V^ concentrations are determined with a linear combination fit from the two extrema spectra. This can be used in a Nernst plot as log([Np^VI^/Np^V^]) plotted as a function of the applied potential (Fig. 3 ▸). Both data sets from the oxidation (red) and reduction (blue) experiments are well aligned within error bars. This evidences the reversibility of the Np^VI^/Np^V^ redox system, confirming that the electrochemical setup operates as expected. From the Nernst plot, a formal potential of the Np^VI^/Np^V^ redox couple in 1 *M* NHO_3_ is determined by linear regression (Fig. 3 ▸, red and blue dotted lines), according to 

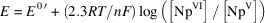

where *E* is the potential, *E* ^0^′ is the apparent standard potential, *R* is the perfect gas constant (J mol^−1^ K^−1^), *T* is temperature (K), *n* is the number of electrons in the reaction, *F* is the Faraday constant and [Np^VI^] and [Np^V^] are the relative con­centrations in Np^VI^ and Np^V^, respectively, as determined by the linear combination fit of the neptunium *L*_3_-edge spectra.

The slopes, ranging between 55.5 and 58.2 mV (±8 mV), are in reasonable agreement with a single electron transfer (expected value of 59 mV at *T* = 298 K). The two formal potentials obtained from the oxidation and reduction experiments are 918.7 and 918.4 mV/(Ag/AgCl), respectively. Herein, the Np^VI^/Np^V^ formal potential given with a maximum uncertainty of ±10 mV is in good agreement with similar studies performed in perchloric acid (Soderholm *et al.*, 1999 ▸; Antonio *et al.*, 2001 ▸) or recent work from Chatterjee *et al.* (2017 ▸) in nitric acid using equivalent spectrophotometric methods.

#### 5.1.1. EXAFS analysis

**Figure 4:**
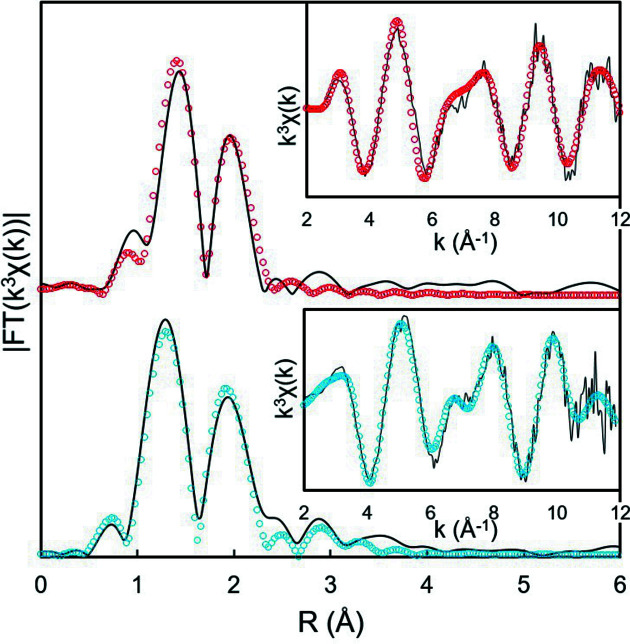
Experimental FT EXAFS signal (line) and best fits (open circles) obtained after 30 min electrolysis at 1150 mV/(Ag/AgCl) (blue) and at 750 mV/(Ag/AgCl) (red). The inset is the corresponding *k* ^3^-weighted EXAFS oscillations.

From electrolysis experiments and analysis of XANES spectra, we demonstrated that the setup provides a fairly robust and reliable method to isolate Np^VI^ and Np^V^ oxidation states by electrolysis. With the potentials set at 1150 and 750 mV/(Ag/AgCl), we expect to stabilize pure species for EXAFS analysis. This controlled potential may stabilize the Np oxidation state upon long EXAFS measurements, thereby balancing for the *in situ* photooxidation due to beam damage. The purpose of the following section is to evaluate whether the electrochemically purified solution results in a more reliable solution to isolate both neptunium oxidation-state ions in a simple solution. In order to compare the results with previous EXAFS measurements on similar systems, we propose to compare the significant fit parameters (CN and DWF) on a single plot. First, Fig. 4 ▸ shows the *k* ^3^-weighted EXAFS oscillations and Fourier transform (FT) for both Np^V^- and Np^VI^-stabilized solutions. Neptunyl ion hydrates show two main oscillations corresponding to two FT peaks typical for hydrated actinyl oxocation (Allen *et al.*, 1997 ▸; Bolvin *et al.*, 2001 ▸; Di Giandomenico *et al.*, 2009 ▸; Duvail *et al.*, 2019 ▸). The structural parameters from simple two oxygen shell EXAFS fits are summarized in Table 1 ▸. The coordination spheres of Np^VI^ and Np^V^ are formed by two O atoms from the neptunyl moiety (O_­yl_) at short *R*_(Np–O­yl)_ distances of 1.75 and 1.82 Å, respectively, and by about 4.4 and 4.9 equatorial O atoms (O_eq_) from water mol­ecules at longer *R*_(Np–Oeq)_ distances of 2.41 and 2.51 Å, respectively. Overall, these results are in line with published values and confirm that a single oxidation state predominates in solution. Distances correspond to the values reported in the literature within a maximum deviation of 0.02 Å (Combes *et al.*, 1992 ▸; Allen *et al.*, 1997 ▸; Antonio *et al.*, 1997 ▸, 2001 ▸; Den Auwer *et al.*, 1999 ▸; Reich *et al.*, 2000 ▸; Bolvin *et al.*, 2001 ▸; Williams *et al.*, 2001 ▸; Kim *et al.*, 2004 ▸; Denecke *et al.*, 2005 ▸; Kim *et al.*, 2005 ▸; Ikeda-Ohno *et al.*, 2008 ▸; Di Giandomenico *et al.*, 2009 ▸; Hennig *et al.*, 2009 ▸; Takao *et al.*, 2009 ▸). However, the hydration numbers (*N*_Oeq_) are known to be much less accurately determined from the EXAFS fits and are still actively discussed. For Np^VI^, the *N*_Oeq_ values from previous EXAFS measurements range between 4.6 and 5.3. For Np^V^, the *N*_Oeq_ values are between 3.6 and 5.2.

The large dispersions reported in the literature are likely due to the mathematical correlation observed in a fitting procedure between the number of scattering atoms in a given shell (*N*), the Debye–Waller factor (σ^2^) and the value of the total amplitude reduction factor ( 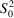 ). Although there is no physical meaning between these physical parameters, upon an EXAFS fit the three parameters *N*, 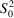 and σ^2^ result in similar effects on the oscilation amplitudes. It is therefore difficult to address coordination numbers from a single EXAFS signal with perfect accuracy, especially if the *k* range is short.

**Figure 5:**
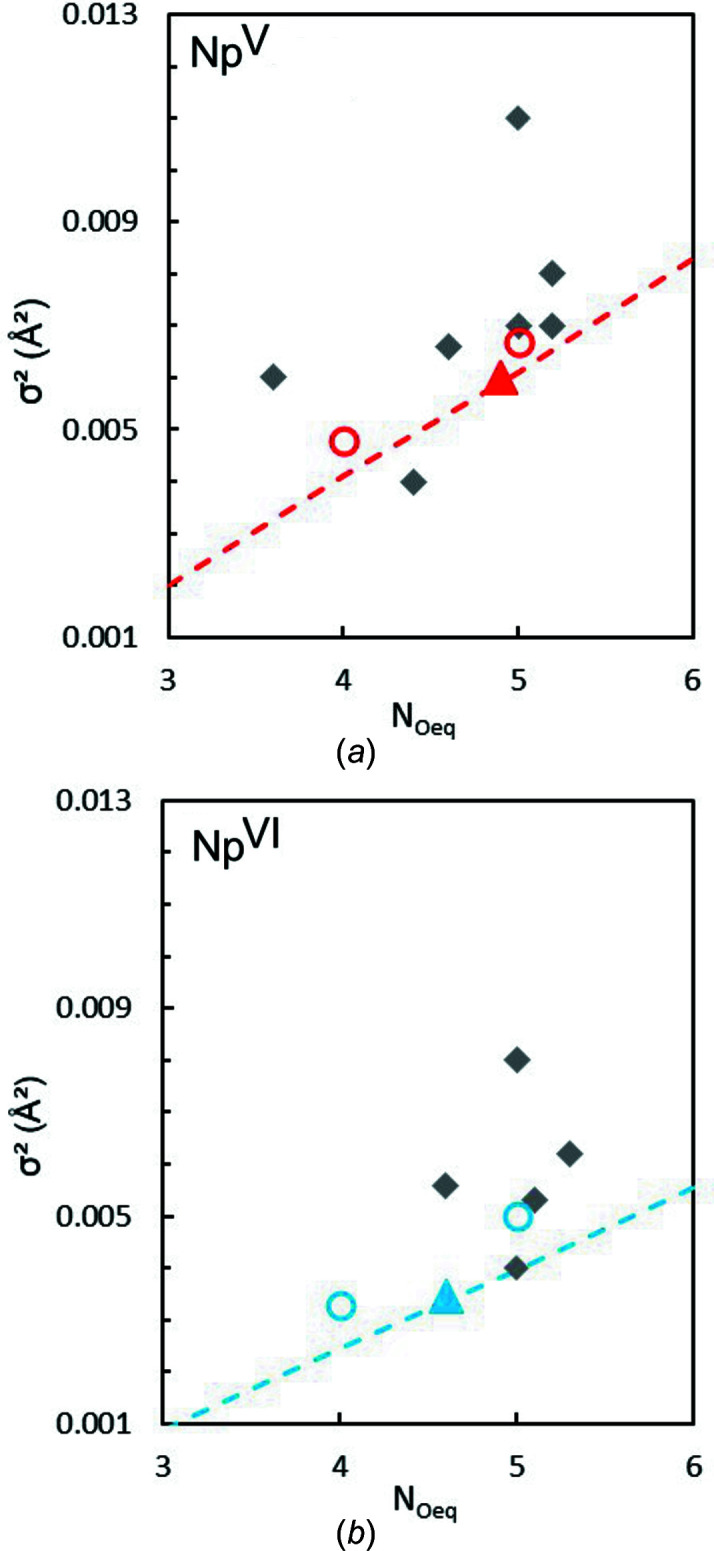
σ^2^ *N* plots from the Np^VI^ and Np^V^ fits. Blue and red dashed lines are the σ^2^ variation as a function of *N*_Oeq_ for Np^VI^ and Np^V^, respectively. Previous reported single-point fit values are given as grey diamonds (Antonio *et al.*, 2001 ▸; Ikeda-Ohno *et al.*, 2008 ▸, 2009 ▸; Hennig *et al.*, 2009 ▸; Di Giandomenico *et al.*, 2009 ▸; Combes *et al.*, 1992 ▸; Allen *et al.*, 1997 ▸; Reich *et al.*, 2000 ▸; Scheinost *et al.*, 2016 ▸; Denecke *et al.*, 2005 ▸). The open circles are DFT-calculated parameters.

In order to estimate the magnitude of these correlations and allow a good comparison between previous EXAFS fits performed under different conditions (*i.e.* fixed or floating CN values and σ^2^), a more complete EXAFS data analysis is proposed hereafter. This analysis follows a method proposed by Ikeda-Ohno *et al.* (2008 ▸). *N*_Oeq_ is successively fixed from 3 to 6 and, for each coordination number step, a best fit is performed. This results in the determination of a conditional σ^2^ variation as a function of *N*. The results can be displayed on a σ^2^ *N* plot, as proposed by Ikeda-Ohno *et al.* (2008 ▸) (Fig. 5 ▸). The best *N*_Oeq_ and σ^2^ combinations (corresponding to Table 1 ▸; values for Np^V^ and Np^VI^) are plotted as triangles in Fig. 5 ▸.

For Np^V^ (Fig. 5 ▸, red dashed line) and Np^VI^ (Fig. 5 ▸, blue dashed line) the σ^2^ values were found to vary approximately linearly with *N*_Oeq_. The Np^V^ trend is similar to that reported by Ikeda-Ohno *et al.* (2008 ▸) for Np^V^. The corresponding *R* factors follow a parabolic variation (not shown) with a minimum at *N*_Oeq_ = 4.9 for Np^V^ and a minimum at *N*_Oeq_ = 4.6 for Np^VI^. However, for both Np^VI^ and Np^V^, reasonable fit parameters were obtained for all tested *N*_Oeq_ values (*i.e. R*_F_ < 5% and σ^2^ < 0.015 Å^2^). This is the reason why this semi-empirical methodology was applied to compare the data with previous results whatever the method used to fit the data. Since the fit can be performed assuming multiple *N*_Oeq_ values, a relevant comparison with any single data point from previous reports needs to be achieved toward the full *N*_Oeq_ range.

On Fig. 5 ▸, the grey diamonds are previous fit parameters determined under similar chemical conditions (*i.e.* noncomplexing and acidic solution). Both *ex situ* measurements (*i.e.* chemical preparation) (Hennig *et al.*, 2009 ▸; Ikeda-Ohno *et al.*, 2008 ▸; Di Giandomenico *et al.*, 2009 ▸; Allen *et al.*, 1997 ▸; Reich *et al.*, 2000 ▸; Combes *et al.*, 1992 ▸) and *in situ* measurements by spectroelectrochemistry (Antonio *et al.*, 2001 ▸) are compared with Np^V^ and Np^VI^ fit parameters from this work. For a given *N*_Oeq_ value, the σ^2^ values (dashed blue and red lines) determined in this work are comparable with or lower than previously published σ^2^ parameters. This is a good indication for a lower disorder/higher purity in this work where Np^V^ and Np^VI^ were purified electrochemically.

Concomitantly, a comparison of the Np^V^ and Np^VI^ structures is in agreement with the expected changes in the neptunyl ion coordination. The σ^2^ values tend to be lower for Np^VI^ than for Np^V^ for a given *N*_Oeq_. This result agrees well with the decrease in atomic charge and bonding strength between water and neptunyl units from Np^VI^ to Np^V^ (Choppin & Rao, 1984 ▸; Denning, 2007 ▸).

At this stage, we conclude that the sample preparation, as well as the continuous application of electrochemical potential, is a good way to maintain a pure oxidation state during X-ray measurements resulting in a low conformational σ^2^. However, this better oxidation-state purification does not solve the difficult question of the hydration structure of actinyl ions in solution. To evaluate this point further, additional information may be extracted to narrow down the actual *N*_Oeq_ values. With this same aim, Ikeda-Ohno *et al.* (2008 ▸) suggested that the *N*_Oeq_ values be derived from the more accurately determined inter­atomic distances applying the bond valence model (Brown, 1978 ▸). Although this approach proved to be satisfying for the well-known coordination chemistry of U^VI^, application to Np is plagued by the limited number of crystallographic references and by uncertainties in the valence unit and the charges of the neptunyl moieties, so that *N*_Oeq_ values still range between 4 and 6. Another way to constrain the accurate coordination number is to associate EXAFS analysis with quantum chemical calculations (Vila *et al.*, 2012 ▸). For some actinide mol­ecular compounds, it is possible to generate EXAFS metrical parameters from a DFT calculation (Acher *et al.*, 2016 ▸, 2017 ▸; Dalodière *et al.*, 2018 ▸). The calculation provides distances and σ^2^ values *ab initio* from a selected structure. The NpO_2_(H_2_O)_4_(H_2_O)_8_^2+^, NpO_2_(H_2_O)_5_(H_2_O)_10_^2+^, NpO_2_(H_2_O)_4_(H_2_O)_8_^+^ and NpO_2_(H_2_O)_5_(H_2_O)_10_^+^ complexes in the presence of a continuum solvent model were selected to produce the parameters reported in Table 2 ▸. The accuracy of the optimized geometry was checked by direct comparison of Np—O_eq_ bond lengths with the measurements. This type of calculation provides an estimated value for the thermal σ^2^ for a given coordination number and for both Np^V^ and Np^VI^. It was possible to compare it with the experimental fit values in Fig. 5 ▸ (red and blue open circles). For both Np^VI^ and Np^V^, inter­estingly, σ^2^ follows the same trend as already observed for experimental fit parameters. However, this trend is obviously not due to the correlation of EXAFS fit parameters since the two values (CN and σ^2^) are determined independently. This inquires a physical inter­relation between a bonding strength and the actinyl coordination number in actinyl solvation. This effect fundamentally impedes the direct determination of actinyl solvatation using the amplitude of the equatorial scattering shell in the EXAFS signal. Again, using distances from both EXAFS and DFT to discriminate between penta- and tetra­hydrated neptunyl ions seems a more reliable method. For Np^V^ with five water mol­ecules, both the calculation and the fit converge for a 2.51 Å bond. For Np^VI^ with five water mol­ecules, 2.41 and 2.42 Å bond lengths are obtained from the fit and the calculation, respectively. Overall, the results support the fact that five water mol­ecules coordinate both the Np^VI^ and the Np^V^ ions.

### 5.2. Np^V^/Np^IV^

**Figure 6:**
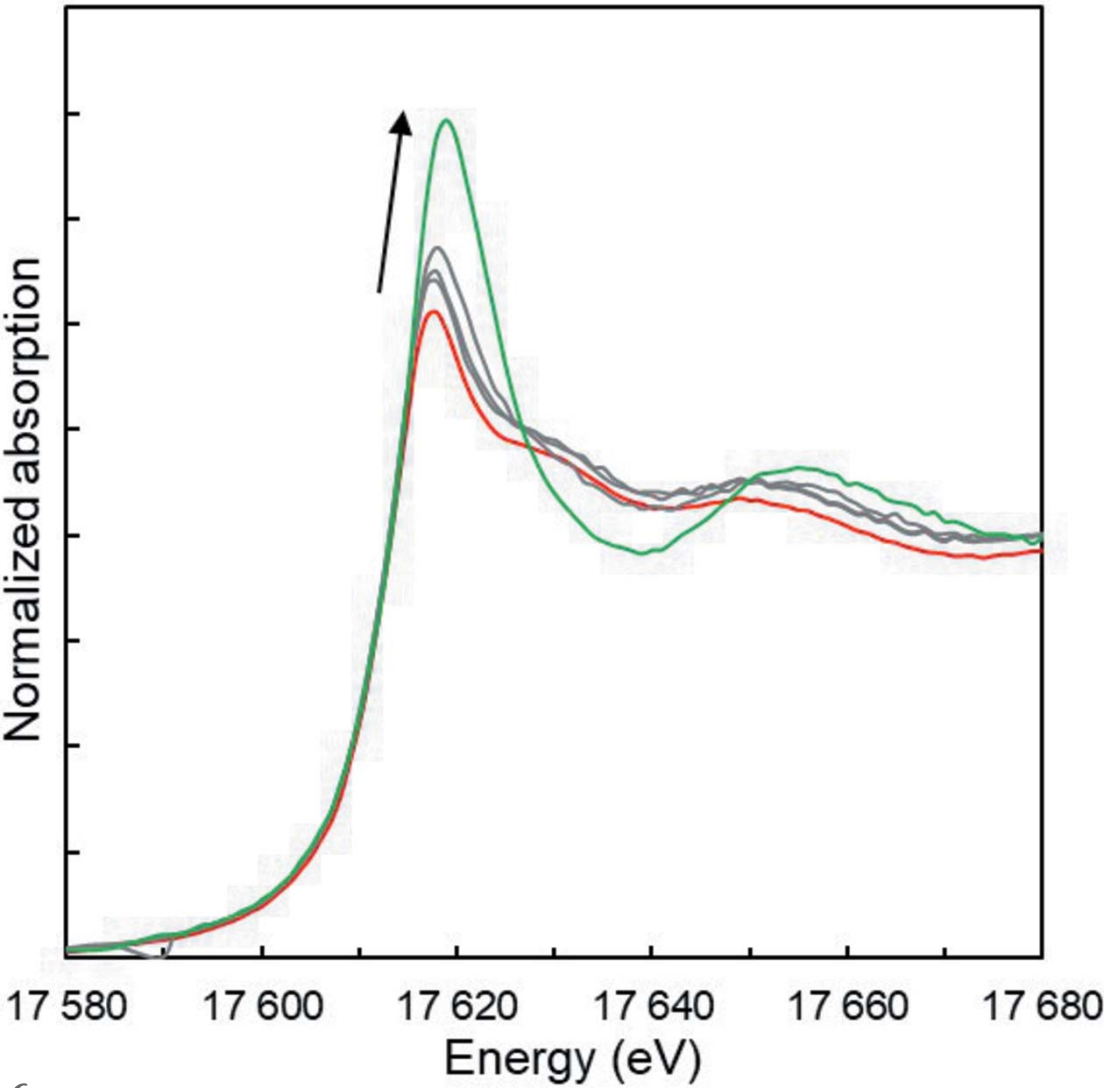
Normalized Np *L*_3_-edge XANES spectrum for the Np^V^ reduction experiment at 750, 0, −100, −150, −200 and −250 mV/(Ag/AgCl).

To evaluate further the electrochemistry setup, a second electrolysis experiment was performed following the previous one. After stabilizing the +V oxidation state by a 1 h electrolysis at 750 mV/(Ag/AgCl), the Np^V^ purity was checked again using XANES and EXAFS. Next, the Np^V^ reduction was investigated by a stepwise decrease of the applied potential to 0, −100, −150, −200 and −250 mV/(Ag/AgCl). In HNO_3_ solution, the Np^IV^ ion is difficult to stabilize due to its reoxidation with HNO_2_ in equilibrium with nitrate ions (Taylor *et al.*, 1998 ▸; Koltunov *et al.*, 1997 ▸). The corresponding XANES spectra are displayed on Fig. 4 ▸. Consistent with previous XANES measurements, the Np *L*_3_-edge drastically changes due to cleavage of the two Np—O bonds in the neptunyl moiety (Bonin *et al.*, 2009 ▸; Denecke *et al.*, 2005 ▸; Hennig *et al.*, 2009 ▸; Scheinost *et al.*, 2016 ▸). Actinide reduction from +V to +IV results in an increase of the white line intensity and a decrease in the amplitude of the higher-energy shoulder at 17637 eV. However, the edge inflection point is almost stabilized by the reduction. XANES spectra are consistent with previous *ex situ* studies and indicate an almost complete reduction of Np^V^ to Np^IV^. More unexpected is the corresponding potential at which this electroreduction occurred. As shown in Fig. 6 ▸, it was mostly Np^V^ that was detected in solution at the potential step of 0 mV. At −100 and −150 mV, the spectra indicate mixed Np^V^/Np^IV^ contributions. Qu­anti­tative Np^V^ reduction is eventually achieved at −200 and −250 mV.

According to recent work (Chatterjee *et al.*, 2017 ▸), reduction to Np^IV^ is expected to occur at approximately 50 mV/(Ag/AgCl) in 1 *M* HNO_3_. Moreover, one would also expect a reduction to the Np^III^ oxidation state below −100 mV/(Ag/AgCl). The Np^IV^/Np^III^ redox potential was determined at approximately −50 mV (Guillaumont *et al.*, 2003 ▸). In such a case, the resulting neptunium *L*_3_ spectra would be a combination of the two redox species. However, Np^III^ is unstable under aerobic conditions and only Np^IV^ seems to be present in the solution after this enforced electrolysis (Hindman *et al.*, 1949 ▸; Sjoblom & Hindman, 1951 ▸; Sullivan *et al.*, 1976 ▸; Cohen, 1976 ▸).

#### 5.2.1. EXAFS analysis

**Figure 7:**
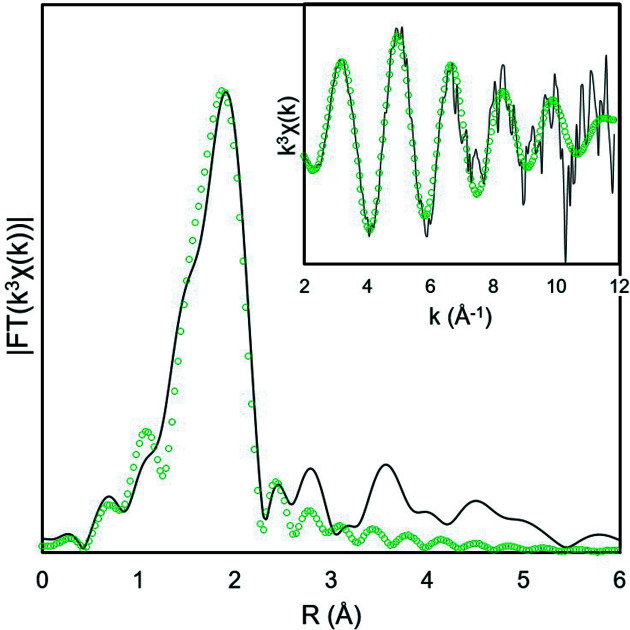
Experimental *k* ^3^-weighted EXAFS oscillations (line) and best fits (open circles) obtained after 30 min electrolysis at −250 mV (green).

From electrolysis experiments and analysis of XANES spectra, we demonstrated that it is possible to obtain Np^IV^ by electrolysis at −250 mV. The *k* ^3^-weighted Np *L*_3_-edge EXAFS spectrum of Np^IV^ hydrate shows a single-frequency oscillation (Fig. 7 ▸) and the corresponding FT displays a single peak which can be reliably modelled with a single Np—O coordination shell. The structural parameters from this fit are summarized in Table 3 ▸. The best fit values correspond to approximately 9.5 O atoms (from water mol­ecules), with an average *R*_(Np–O)_ distance of 2.39 Å.

The Np—O distance corresponds to the values reported in the literature of 2.37–2.41 Å (Combes *et al.*, 1992 ▸; Allen *et al.*, 1997 ▸; Reich *et al.*, 2000 ▸; Williams *et al.*, 2001 ▸; Bolvin *et al.*, 2001 ▸; Antonio *et al.*, 2001 ▸). The *N*_H2O_ value also falls within the range of previous EXAFS results for Np^IV^ aqua complexes, *i.e.* between 8.7 and 11.6 (Combes *et al.*, 1992 ▸; Allen *et al.*, 1997 ▸; Reich *et al.*, 2000 ▸; Williams *et al.*, 2001 ▸; Bolvin *et al.*, 2001 ▸; Antonio *et al.*, 2001 ▸).

**Figure 8:**
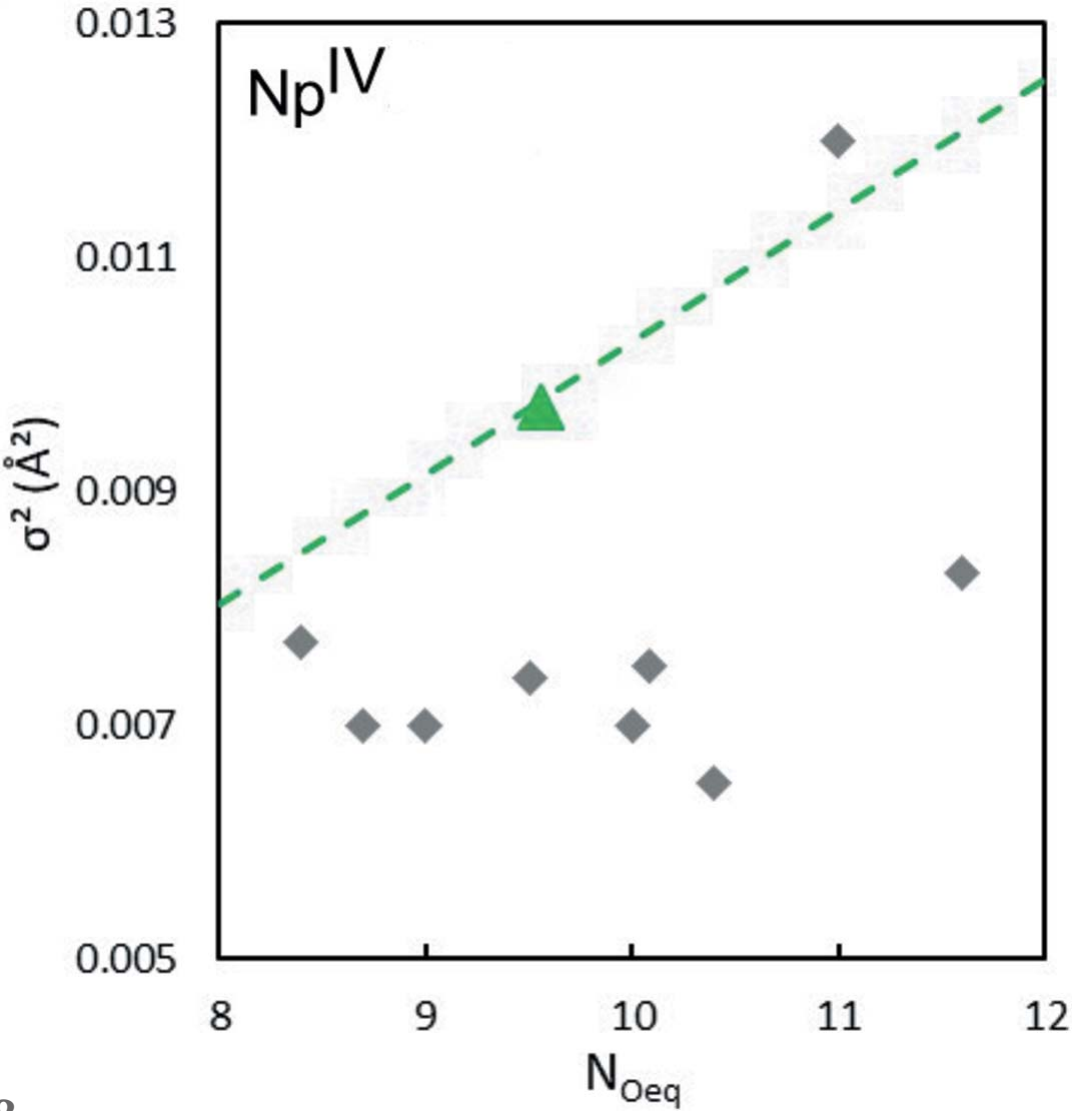
Plot of the σ^2^ values as a function of coordination number from the EXAFS fit (dashed line) and comparison with previously reported values (Antonio *et al.*, 2001 ▸; Ikeda-Ohno *et al.*, 2008 ▸, 2009 ▸; Hennig *et al.*, 2009 ▸; Di Giandomenico *et al.*, 2009 ▸; Combes *et al.*, 1992 ▸; Allen *et al.*, 1997 ▸; Reich *et al.*, 2000 ▸; Scheinost *et al.*, 2016 ▸; Denecke *et al.*, 2005 ▸) (grey diamonds). The triangle shows the best fit results from this work.

As for Np^V^ and Np^VI^, a clear correlation between σ^2^ and *N*_O_ can be drawn with restricted *N*_O_ fits. Fixing *N*_O_ from 8 to 11 allows the determination of the corresponding σ^2^. These values approximately align as a function of *N*_O_ (Fig. 8 ▸, green dashed line), together with the best fit values (triangle). The corresponding data determined previously on analogous systems are compared (grey diamond). Inter­estingly, almost all previous studies report a lower σ^2^ value for a given *N*_O_ compared with the present report. The more distorted geometry of the 8 to 11 water mol­ecule coordination shell results in σ^2^ values (from 0.0065 to 0.012 Å^2^) much larger than observed for the neptunyl hydration shell. This time the comparison with previous studies clearly indicates a residual disorder in the neptunium coordination sphere for the *in situ* prepared Np^4+^. This may be explained by either (i) a residual NpO_2_^+^ contribution in the EXAFS spectrum, (ii) over-reduction resulting in the formation of Np^III^ with a coordination shell at about 2.5 Å (Antonio *et al.*, 2001 ▸; Brendebach *et al.*, 2009 ▸) or (iii) complexation of Np^IV^ by nitrate (by analogy with Pu^IV^ chemistry in nitric acid this must be quite insignificant; Allen *et al.*, 1996 ▸), or a combination of points (i), (ii) and (iii).

## 6. Conclusions

The main aim of this study was to develop a spectroelectrochimical cell and to qualify this setup for further applications on radioactive samples. During the experiment, it was possible to investigate the neptunium coordination during redox processes in a simple 1 *M* HNO_3_ solution.

The technical parameters of the microcell design are presented herein and its first application to transuranic elements, performed safely, are reported. The cell design minimizes the volume of radioactive solution and facilitates transport and handling on the beamline. Low actinide concentration and volume minimize the sample activity and allows XANES and EXAFS measurements within a reasonable timescale. From an electrochemistry point of view, such a static confinement concept is not ideal. On one hand, cycling the NpO_2_^2+^/NpO_2_^+^ redox couple was quite possible and resulted in reliable standard potential determination. It was then possible to measure both Np^V^ and Np^VI^ EXAFS spectra under fully consistent conditions. This was a good opportunity to compare the Np^V^ and Np^VI^ hydration spheres, and to implement a statistical comparison with formal literature results, as well as theoretical models. The evolution in the equatorial actinyl hydration is well characterized and is consistent with theoretical expectations. Moreover, a detailed comparison with previous structural data using EXAFS established that the stabilization of Np^V^ and Np^VI^ oxidation by *in situ* electrochemistry appears to be the most reliable way to maintain a pure oxidation state during X-ray measurements. On the other hand, the reduction to Np^IV^ never fully succeeded in maintaining pure Np^IV^ in solution. From the point of view of electrochemistry, a clear offset in the expected standard potential must be applied to begin the Np^V^ reduction. The resulting Np^IV^ EAXFS spectra reveal hydrated Np^4+^ as the main species, but comparison with a previous chemically stabilized Np^IV^ solution indicates a more disordered coordination sphere. Altogether, it seems that, while the results are fully satisfying for the oxidized neptunium species, the cell must be used with care to study reduced neptunium forms. As a final point, this cell, primarily designed to confine radioactive samples for user safety, was also efficient for performing measurements on nonradioactive samples. Its design for radioactive samples appears to be versatile and extremely convenient for preventing moisture and oxygen contaminating air-sensitive samples in the reverse direction. By doing so, the *in situ* XAS stabilization and characterization of Ln^II^ in room-temperature ionic liquid samples was made possible with this cell (Bengio *et al.*, 2018 ▸, 2020 ▸) and other applications of this kind are expected.

## Figures and Tables

**Table 1:** Metrical parameters from EXAFS fits Asterisks (*) indicate fixed parameters.

	Path	*N*	*R*	σ^2^
Np^VI^ Δ*E*_0_ 5 eV*R* factor 2.1%	O_­yl_	2*	1.75 (1)	0.0022 (10)
	O_H2O_	4.6 (7)	2.41 (2)	0.0035 (15)

Np^V^ Δ*E*_0_ −1 eV*R* factor 1.8%	O_­yl_	2*	1.82 (1)	0.0019 (10)
	O_H2O_	4.9 (6)	2.51 (2)	0.0060 (15)

**Table 2:** DFT calculations of Np—O bond distances (average values in Å) and Debye–Waller factor σ^2^ (Å^2^) in Np^V^ and Np^VI^ hydrated clusters with four and five coordinated water mol­ecules

	Np—O_­yl_	σ^2^	Np—O_wat_	σ^2^
Np^V^(H_2_O)_4_(H_2_O)_8_^+^	1.80	0.0014	2.47	0.0048
Np^V^(H_2_O)_5_(H_2_O)_10_^+^	1.82	0.0016	2.51	0.0068
Np^VI^(H_2_O)_4_(H_2_O)_8_^2+^	1.73	0.0012	2.32	0.0033
Np^VI^(H_2_O)_5_(H_2_O)_10_^2+^	1.73	0.0013	2.42	0.0050

**Table 3:** Parameters from Np^IV^ EXAFS fits Asterisks (*) indicate fixed parameters.

	Path	CN	*R*	σ^2^
S_{0}^{\,2} 1* Δ*E*_0_ −4 eV *R* factor 3.8%	O_H2O_	9.5 (16)	2.39 (2)	0.0095 (15)
